# Selenite Bioremediation by Food-Grade Probiotic Lactobacillus casei ATCC 393: Insights from Proteomics Analysis

**DOI:** 10.1128/spectrum.00659-23

**Published:** 2023-05-23

**Authors:** Lei Qiao, Xina Dou, Xiaofan Song, Jiajing Chang, Xiaonan Zeng, Lixu Zhu, Chunlan Xu

**Affiliations:** a School of Life Sciences, Northwestern Polytechnical University, Xi’an, Shaanxi, China; University of Minnesota Twin Cities

**Keywords:** ABC transporter, glutathione, Painter-type reaction, selenite reduction, selenium nanoparticles

## Abstract

Microorganisms capable of converting toxic selenite into elemental selenium (Se^0^) are considered an important and effective approach for bioremediation of Se contamination. In this study, we investigated the mechanism of reducing selenite to Se^0^ and forming Se nanoparticles (SeNPs) by food-grade probiotic Lactobacillus casei ATCC 393 (*L. casei* ATCC 393) through proteomics analysis. The results showed that selenite added during the exponential growth period of bacteria has the highest reduction efficiency, and 4.0 mM selenite decreased by nearly 95% within 72 h and formed protein-capped-SeNPs. Proteomics analysis revealed that selenite induced a significant increase in the expression of glutaredoxin, oxidoreductase, and ATP binding cassette (ABC) transporter, which can transport glutathione (GSH) and selenite. Selenite treatment significantly increased the *CydC* and *CydD* (putative cysteine and glutathione importer, ABC transporter) mRNA expression level, GSH content, and GSH reductase activity. Furthermore, supplementation with an additional GSH significantly increased the reduction rate of selenite, while GSH depletion significantly inhibited the reduction of selenite, indicating that GSH-mediated Painter-type reaction may be the main pathway of selenite reduction in *L. casei* ATCC 393. Moreover, nitrate reductase also participates in the reduction process of selenite, but it is not the primary factor. Overall, *L. casei* ATCC 393 effectively reduced selenite to SeNPs by GSH and nitrate reductase-mediated reduction pathway, and the GSH pathway played the decisive role, which provides an environmentally friendly biocatalyst for the bioremediation of Se contamination.

**IMPORTANCE** Due to the high solubility and bioavailability of selenite, and its widespread use in industrial and agricultural production, it is easy to cause selenite to accumulate in the environment and reach toxic levels. Although the bacteria screened from special environments have high selenite tolerance, their safety has not been fully verified. It is necessary to screen out strains with selenite-reducing ability from nonpathogenic, functionally known, and widely used strains. Herein, we found food-grade probiotic *L. casei* ATCC 393 effectively reduced selenite to SeNPs by GSH and nitrate reductase-mediated reduction pathway, which provides an environmentally friendly biocatalyst for the bioremediation of Se contamination.

## INTRODUCTION

Selenium (Se), as an indispensable micronutrient element for the body, participates in many important physiological functions, including antioxidant, immune regulation, and antiviral, by being incorporated into selenoproteins (1). Se is reported to be one of the chemical elements with the narrowest range between dietary deficiency (<40 μg/day) and toxicity (>400 μg/day) (2, 3). Long-term Se deficiency can lead to endemic Keshan disease, intestinal disorders, and epilepsy (4). However, excessive Se intake can cause damage to the nervous, immune, cardiovascular, and reproductive systems of vertebrates (5). Therefore, it is necessary to carefully control the intake of Se by humans and other animals. Se occurs naturally in large quantities in ores, sedimentary rocks, and soils in volcanic regions and can be released into the environment through weathering (3, 6). In addition, human-related activities such as industrial and agricultural production, oil burning, and metal extraction also increase environmental fluxes of Se, reaching hazardous levels in water and soil and potentially entering the food chain (7, 8). Furthermore, it is worth noting that the toxicity of Se depends not only on the concentration but also on the chemical form. Se exists in nature in many forms, including selenate (Se [VI]), selenite (Se [IV]), elemental Se (Se [0]), selenide (Se [–II]), selenomethionine, and selenocysteine/selenoprotein (9, 10). The toxicity of Se species is ranked as follows: selenite > selenocysteine > selenate ≈ selenomethionine > elemental Se (11). Due to the high solubility and bioavailability of selenite, and its widespread use in industrial and agricultural production, it is easy to cause selenite to accumulate in the environment and reach toxic levels (12, 13).

Therefore, it is urgent to develop an efficient, low-cost, and environmentally friendly method to remediate Se contamination.

Microorganisms fundamentally contribute to the biogeochemical cycle of Se by altering its chemical state, thereby determining the fate and mobility of Se (14). In recent years, numerous bacterial strains have been studied for their capacity to reduce selenite to immobile and less toxic Se^0^ and form Se nanoparticles (SeNPs), through assimilatory or dissimilatory metabolism, or detoxification processes (15). Stenotrophomonas bentonitica BII-R7 has been shown to effectively reduce soluble Se in its selenite form to insoluble and less toxic Se^0^ (14). Huang et al. (16) reported a highly selenite-resistant strain Providencia rettgeri HF16 that completely transformed 5 mM selenite within 24 h and detected SeNPs as early as 2 h. In addition, a highly selenite-resistant strain Proteus penneri LAB-1, isolated from lateritic red soil, reduced selenite by nearly 2 mM within 18 h and produced SeNPs at the beginning of the exponential phase (17). Fischer et al. (18) isolated Bacillus safensis JG-B5T from the soil in a uranium mining waste pile that reduced 2.5 mM selenite by 70% and produced red spherical SeNPs extracellularly. In engineered systems, biological treatment of Se-contaminated wastewater is the preferred removal technique (19). However, the safety of some strains has not been fully verified, and if it spills into the environment during the application process, it may harm the environment and cause risks such as human and animal infection. In addition, most of the SeNP-synthesizing strains currently studied are Gram-negative bacteria whose outer membranes contain lipopolysaccharide (LPS), which prevents their further utilization (9). Finding safe, nonpathogenic bacteria for bioremediation of selenite contamination is highly valuable. Avendaño et al. (20) found that Pseudomonas putida KT2440 (a safe nonpathogenic bacterium), which is commonly utilized for environmental purposes, exhibits remarkable efficacy in selenite reduction while demonstrating superior resistance to oxidative stress in comparison to other bacterial strains, making it more suitable for the selenite reduction process. Vibrio natriegens is also a nonpathogenic, rapidly growing bacterium (doubling time less than 10 min) that exhibits remarkable resistance to selenite, capable of rapid reduction of selenite to Se^0^ (21). In addition, Thauera selenatis is the most extensively researched selenate-respiring bacterium and has been utilized for the bioremediation of Se-contaminated agricultural drainage water. Widespread use of Thauera selenatis could reduce the amount of Se (selenate, selenite) deposited in the San Joaquin River from 3,000 to 60 kg/year (22).

As a generally recognized as safe (GRAS) microorganism, probiotics have the characteristics of nonpathogenicity, rapid growth, and can also produce a variety of proteins and enzymes involved in selenite reduction and SeNPs synthesis (23). Li et al. (24) investigated the ability of eight lactic acid bacteria (LAB) to tolerate and convert selenite and found that the Lactobacillus paralimentarius strain JZ07 exhibited high levels of selenite resistance and biotransformation. Rajasree and (25) Gayathri reported that using *Lactobacillus* spp. can reduce sodium selenite to spherical SeNPs with sizes ranging from 20 to 150 nm and deposit them in the intracellular region. In addition to lactic acid bacteria, Bacillus subtilis, Enterococcus faecium, Saccharomyces cerevisiae also have the ability to reduce selenite to synthesize SeNPs (26–28). The above research provides an effective and harmless bioremediation strategy for the remediation of Se contamination. Moreover, the intracellular synthesis of SeNPs can effectively prevent the rediffusion of Se into the environment, which can be used by aquatic organisms and cause poisoning (6).

More importantly, SeNPs have received extensive attention due to their biological properties such as low toxicity and high biocompatibility and are considered a safer and more effective Se nutritional supplement (1). In addition, the unique optoelectronic, semiconducting, photoconductive, and catalytic properties of Se make it suitable for a wide range of applications in the electronics and optics industries (16). The characteristic of probiotics to synthesize SeNPs by reducing selenite is like killing two birds with one stone. It cannot only harmlessly repair Se contamination, but also can be used as a biological factory to synthesize SeNPs for biomedicine or industrial biomaterials.

Lactobacillus casei (Gram-positive bacteria) is one of the most widely studied and applied LABs, and its health-promoting ability has been confirmed in many studies (29, 30). We initially found that food-grade Lactobacillus casei ATCC 393 (*L. casei* ATCC 393) can reduce sodium selenite to synthesize SeNPs (31), but its reduction efficiency and mechanism have not been studied in depth. Therefore, in this study, we mainly studied the reduction efficiency of *L. casei* ATCC 393 to selenite and identified the surface-capped protein of biogenic SeNPs. Moreover, the proteins or enzymes involved in selenite reduction and SeNPs biosynthesis were explored through iTRAQ coupled with LC-MS/MS. Here, the expression of key proteins and the activities of key enzymes were further investigated to verify the proposed mechanism of reducing selenite to Se^0^ and form SeNPs.

## RESULTS

### Selenite reduction and elemental Se formation by *L. casei* ATCC 393.

**FIG 1:**
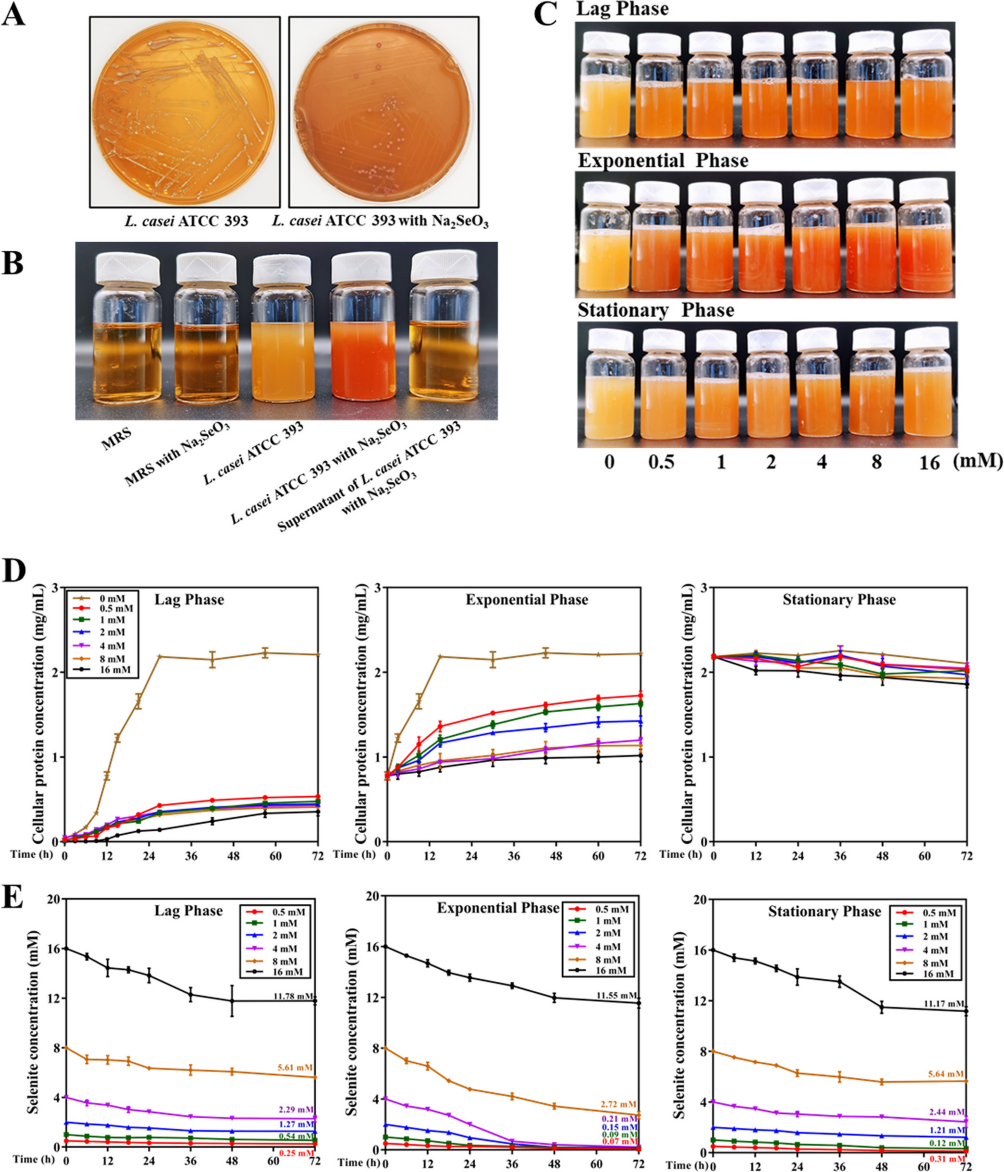
Selenite reduction and elemental Se^0^ formation by *L. casei* ATCC 393. (A) Images of *L. casei* ATCC 393 cultures in the absence (left) and presence (right) of 1 mM sodium selenite on MRS agar plates. (B) Appearance color of MRS broth medium, *L. casei* ATCC 393 supernatant, or *L. casei* ATCC 393 cultivated with or without sodium selenite at 37°C for 48 h. (C) Color change of *L. casei* ATCC 393 cultures supplemented with different concentrations of sodium selenite (0 mM, 0.5 mM, 1 mM, 2 mM, 4 mM, 8 mM, and 16 mM) at different growth stages (lag phase: 3 h, exponential phase: 12 h, stationary phase: 27 h) of incubation. (D) Effects of different concentrations of sodium selenite on bacterial growth at different growth stages of *L. casei* ATCC 393. Samples of 1 mL of the bacterial culture were collected at different time intervals of bacteria growth and then centrifuged at 4°C. Protein was extracted from the pellet using a total bacterial protein extraction assay kit. Bacterial growth was measured via the quantification of total cell protein. The protein concentration in bacteria cell extracts was determined. (E) Effects of different addition time and different concentrations of sodium selenite on the removal of selenite. Samples of 1 mL of the bacterial culture were collected at different time intervals of bacteria growth and then centrifuged at 4°C. After taking the supernatant, ICP-MS was used to detect the Se content to monitor the dynamic change of selenite residue in the medium, and then determine the reduction efficiency of *L. casei* ATCC 393 to selenite. Data are expressed as mean ± SEM. *N* = 3.

In this study, we investigated the potential of food-grade LAB *L. casei* ATCC 393 to efficiently reduce selenite and contribute to the bioremediation of Se contamination. As shown in Fig. 1A, monoclonal bacteria of *L. casei* ATCC 393 appear red after 72 h of culture on De Man Rogosa Sharpe (MRS) agarose medium with 1 mM sodium selenite. Subsequently, the strain was cultured in MRS medium with 1 mM sodium selenite at 37°C without shaking for 48 h. Throughout the cultivation process, we observed that both MRS culture medium and *L. casei* ATCC 393 supernatant, with or without sodium selenite, appeared as transparent deep yellow. However, after being cultivated with 1 mM sodium selenite for 48 h, *L. casei* ATCC 393 exhibited a distinct bright deep-red color (Fig. 1B). The above phenomena suggest that *L. casei* ATCC 393 can convert selenite to elemental Se.

To determine the reduction efficiency of *L. casei* ATCC 393 for selenite, different concentrations (0 mM, 0.5 mM, 1 mM, 2 mM, 4 mM, 8 mM, and 16 mM) of sodium selenite were added to coculture for 72 h at different growth stages of *L. casei* ATCC 393 (lag phase: 3 h, exponential phase: 12 h, stationary phase: 27 h), and the concentration of selenite in the medium was detected. Based on the discernible alterations in the MRS broth medium depicted in Fig. 1C, it was observed that the culture yielded a crimson or vivid red cell suspension, thereby indicating its proficiency in reducing the soluble and colorless selenite ions to insoluble and red elemental Se. It is noteworthy that the hue of the medium grew progressively darker in correlation with the augmentation of selenite concentration. In addition, the medium in the exponential phase of bacteria appeared the darkest red, followed by the lag phase, and the red in the stationary phase was the lightest, which indicated that the addition of sodium selenite at different periods had a great influence on the reduction efficiency of *L. casei* ATCC 393. Moreover, compared with 8 mM sodium selenite, the medium with 16 mM sodium selenite presented a lighter red color, indicating that the higher concentration of selenite had a toxic effect on *L. casei* ATCC 393 and was not conducive to its reduction. By detecting the growth of bacteria, it was found that adding sodium selenite in the lag phase and exponential phase would inhibit the growth of *L. casei* ATCC 393, and it had a dose-dependent effect, and the higher the concentration, the more obvious the inhibitory effect. In the stationary phase, with the increase of culture time, the growth of *L. casei* ATCC 393 showed a downward trend (Fig. 1D). Furthermore, as shown in Fig. 1E, selenite added during the exponential phase was rapidly reduced by *L. casei* ATCC 393, with 4 mM selenite reduced by 95% ± 1% over 72 h. In the exponential phase group, compared with 4 mM sodium selenite, 8 mM and 16 mM sodium selenite showed lower reduction rates, with reduction rates of 66% ± 5% and 28% ± 3%, respectively.

### Localization and characterization of SeNPs.

**FIG 2:**
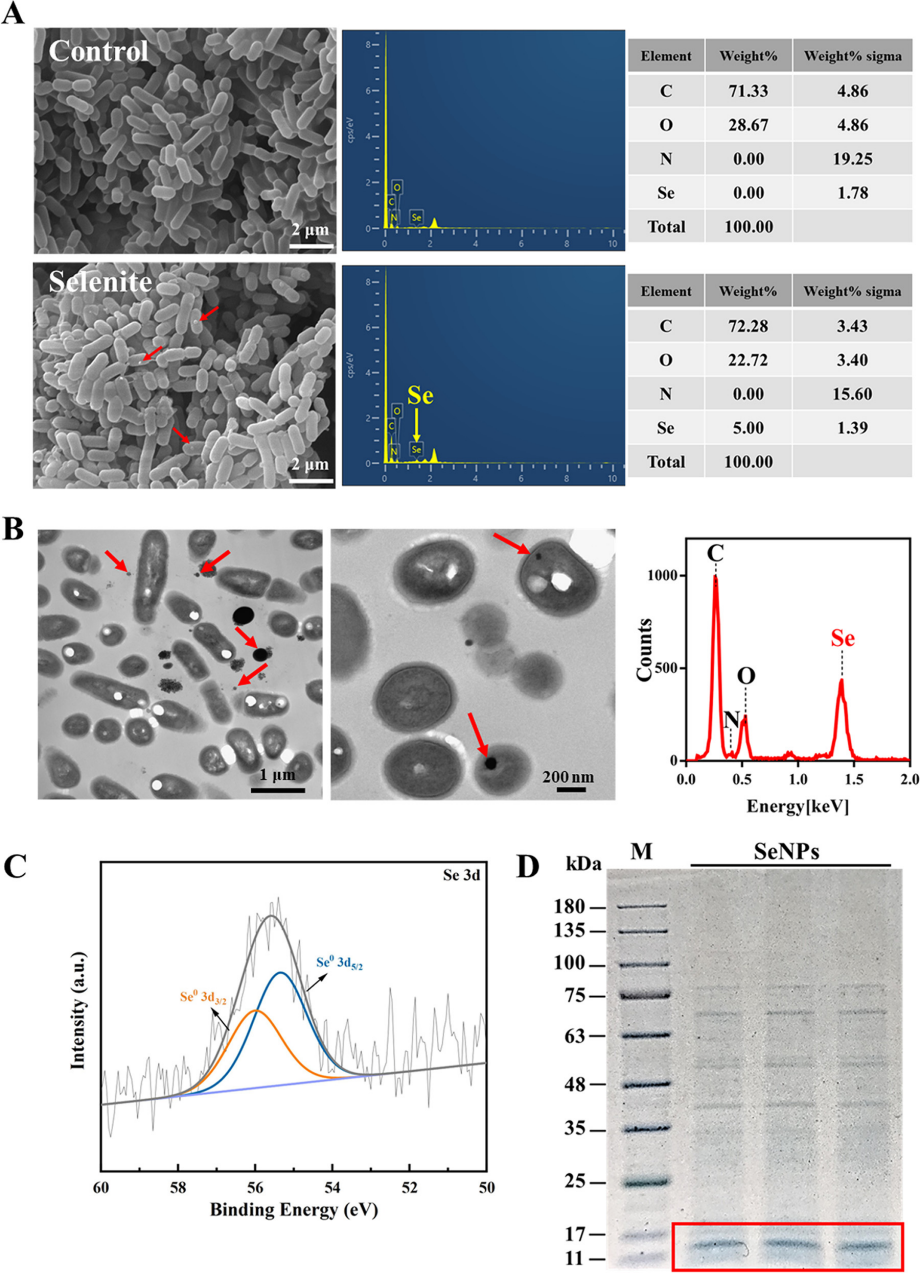
Localization and characterization of *L. casei* ATCC 393 synthesized SeNPs. (A) SEM micrographs of the *L. casei* ATCC 393 cultured in the absence (top) or presence (bottom) of 4 mM sodium selenite for 72 h. EDS analysis of the contents of carbon (C), nitrogen (N), oxygen (O), and Se in the area of micrographs. (B) TEM micrographs of *L. casei* ATCC 393 and SeNPs, and EDX analysis of the elemental composition of SeNPs. (C) High-resolution Se 3D XPS of purified SeNPs synthesized by *L. casei* ATCC 393. (D) SDS-PAGE gel images (Coomassie blue staining) of proteins associated with biogenic proteins-capped SeNPs (M: Marker).

Scanning electron microscope (SEM) analysis revealed that 4 mM sodium selenite treatment had no obvious effect on the morphology of *L. casei* ATCC 393. However, spherical particles in the range of 40 to 200 nm were observed attached to the cells after sodium selenite treatment (Fig. 2A). Energy dispersive spectrometer (EDS) flat scanning of the region containing spherical particles revealed a strong Se atom signal, accounting for 5.00% of the total component elements, suggesting that these particles may be SeNPs (Fig. 2A). In addition, transmission electron microscope (TEM) analysis also confirmed the presence of electron-dense nanoparticles ranging in size from approximately 65 ± 22 nm both inside and outside the cells (Fig. 2B). Further energy dispersive X-ray spectroscopy (EDX) point-scan analysis of nanoparticles and the EDX spectrum clearly showed the presence of Se specific peak. Moreover, Se, carbon (C), oxygen (O), nitrogen (N), and sulfur (S) overlapped in the nanoparticle distribution area (Fig. 2B), suggesting that these nanoparticles may be coated with biomass.

To further confirm the valence state of Se in these Se-containing nanoparticles, we extracted and isolated the nanoparticles from the bacteria and performed XPS analysis. Fig. 2C clearly showed the 3D spectral peaks of Se^0^ (Se^0^ 3d_3/2_ and Se^0^ 3d_5/2_). These results suggested that *L. casei* ATCC 393 reduces selenite to Se^0^ and assembles it into nanoparticles (SeNPs). Moreover, we also analyzed the composition of the biomass on the surface of SeNPs. The previous study (31) found that the surface biomass of SeNPs is mainly protein, so we used LC-MS/MS to identify its surface proteins. As shown in Fig. 2D, the surface of SeNPs is covered with a variety of proteins. Among them, the proteins with a molecular weight of 11 to 17 kDa were abundant. Those proteins were analyzed by LC-MS/MS. Further analysis based on the UniProt database showed that the main protein which capped SeNPs was 50S ribosomal protein L7/L12 (ID: S6BUA7_LACCA) with 12.6 kDa (Table 1). As shown in Fig. S1 in the supplemental material, the Zeta potential value of SeNPs in 0.01 M phosphate-buffered saline (PBS) (pH 7.4) was −37.9 mV, which indicated that the existence of SeNPs surface proteins makes it highly stable in solution and not easy to aggregate. SeNPs with small particle size and high stability could exhibit their intrinsic advantage in biomedicine, agriculture, and biosensor applications.

**TABLE 1:** Proteins were analyzed by LC-MS/MS and the UniProt database

Protein ID	Sequence coverage [%]	mol wt [kDa]	Score
S6C9N0	52.8	57.569	318.12
S6BUA7	93.4	12.617	196.17
S6CAV2	83.7	16.609	160.45
S6CBP1	60.3	14.88	129.28
S6CJS2	27.5	59.586	110.95
S6BXC8	20.4	72.554	107.44
S6C968	39.9	18.854	91.331
S6C617	33.6	16.15	70.471
S6CJM2	69.4	16.136	68.533
S6C000	13.4	12.902	63.619

### Quantitative proteomic analysis of *L. casei* ATCC 393 exposed to selenite.

**FIG 3:**
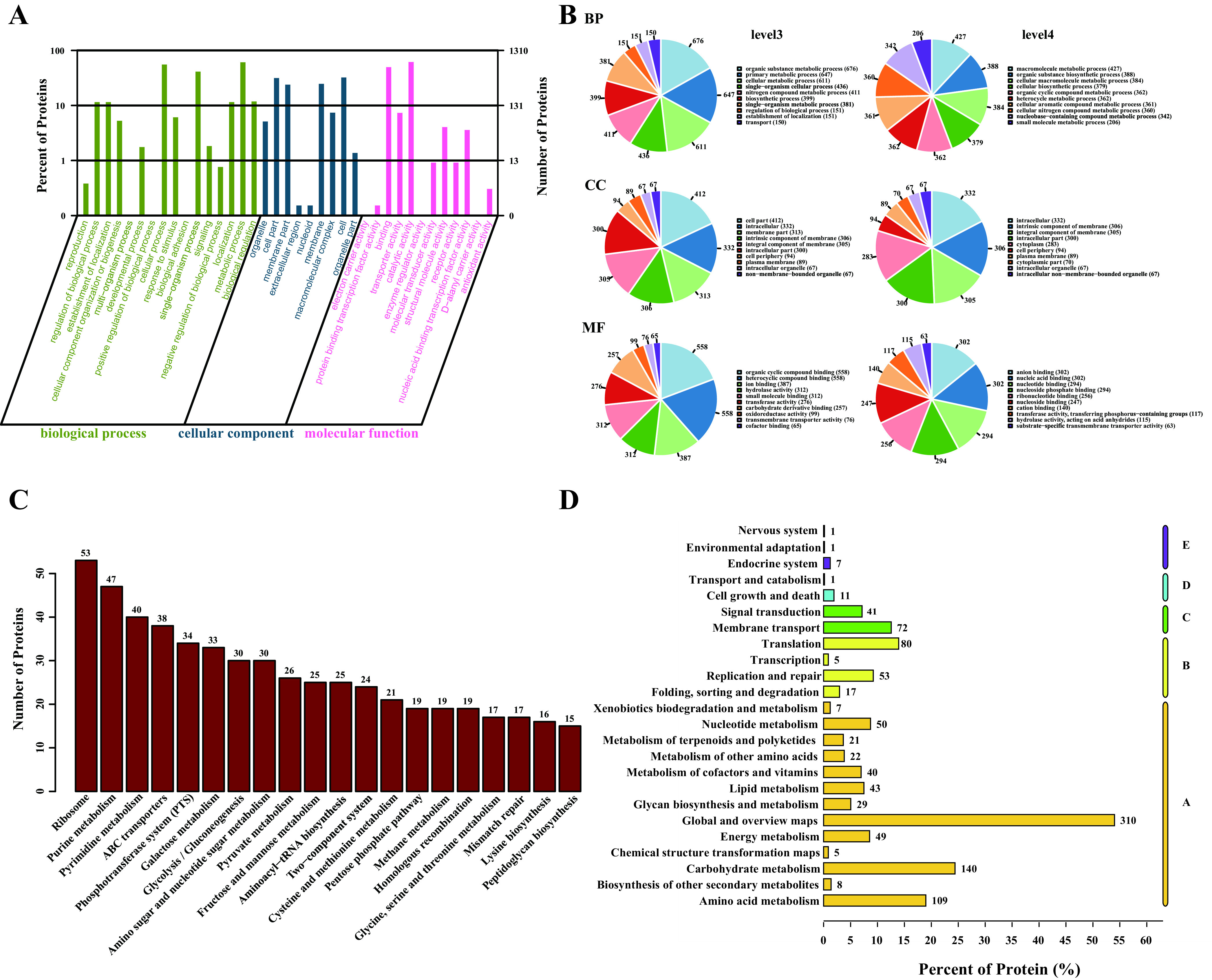
Proteome functional annotation. (A) GO annotation in terms of molecular function at level 2. (B) GO annotation in terms of molecular function at level 3 and level 4. (C) KEGG pathway analyses of protein identified. (D) The top 20 pathways with the most proteins included in the KEGG analysis. BP: biological process, CC: cell component, MF: molecular function, A: metabolism, B: genetic information processing, C: environmental information processing, D: cellular processes, E: organismal systems. *N* = 3.

To gain further insights into the mechanisms underlying the reduction of selenite and the biosynthesis of SeNPs, the iTRAQ-based quantitative proteomic analysis was conducted to investigate the proteomic differences of *L. casei* ATCC 393 treated with or without selenite. A total of 17,002 peptides and 1,703 proteins were detected and identified as belonging to the proteome of *L. casei* ATCC 393 in this study (Fig. S2). Gene Ontology (GO) functional annotation was performed on all quantitatively obtained proteins to explore the biological functions of these proteins. According to Fig. 3A and B, the preponderance of the identified proteins are annotated in catalytic activity, metabolic process, binding, cellular process and single-organism process at level 2 (top five). At the third level, the proteins are categorized based on the frequency of annotations, with the top five being the organic substance metabolic process, primary metabolic process, cellular metabolic process, organic cyclic compound binding, and heterocyclic compound binding. In the level 4 annotation, we found that the identified proteins are mainly involved in biological processes (BP), including macromolecule metabolic, organic substance biosynthetic, cellular macromolecule metabolic, cellular biosynthetic, organic cyclic compound metabolic, heterocycle metabolic, cellular aromatic compound metabolic, cellular nitrogen compound metabolic, and nucleobase-containing compound metabolic. In addition, the KEGG database was used to annotate the identified proteins. As shown in Fig. 3C, most proteins are annotated into metabolism. Fig. 3D shows the top 20 pathways with the largest number of proteins, mainly including ribosome, purine metabolism, pyrimidine metabolism, ATP binding cassette (ABC) transporters, phosphotransferase system, galactose metabolism, pentose phosphate pathway, and so on. Notably, 53 proteins were clustered in the ribosome, and we previously found that the surface of SeNPs was capped by 50S ribosomal protein L7/L12, suggesting that ribosome-related proteins may be involved in the assembly of SeNPs.

**FIG 4:**
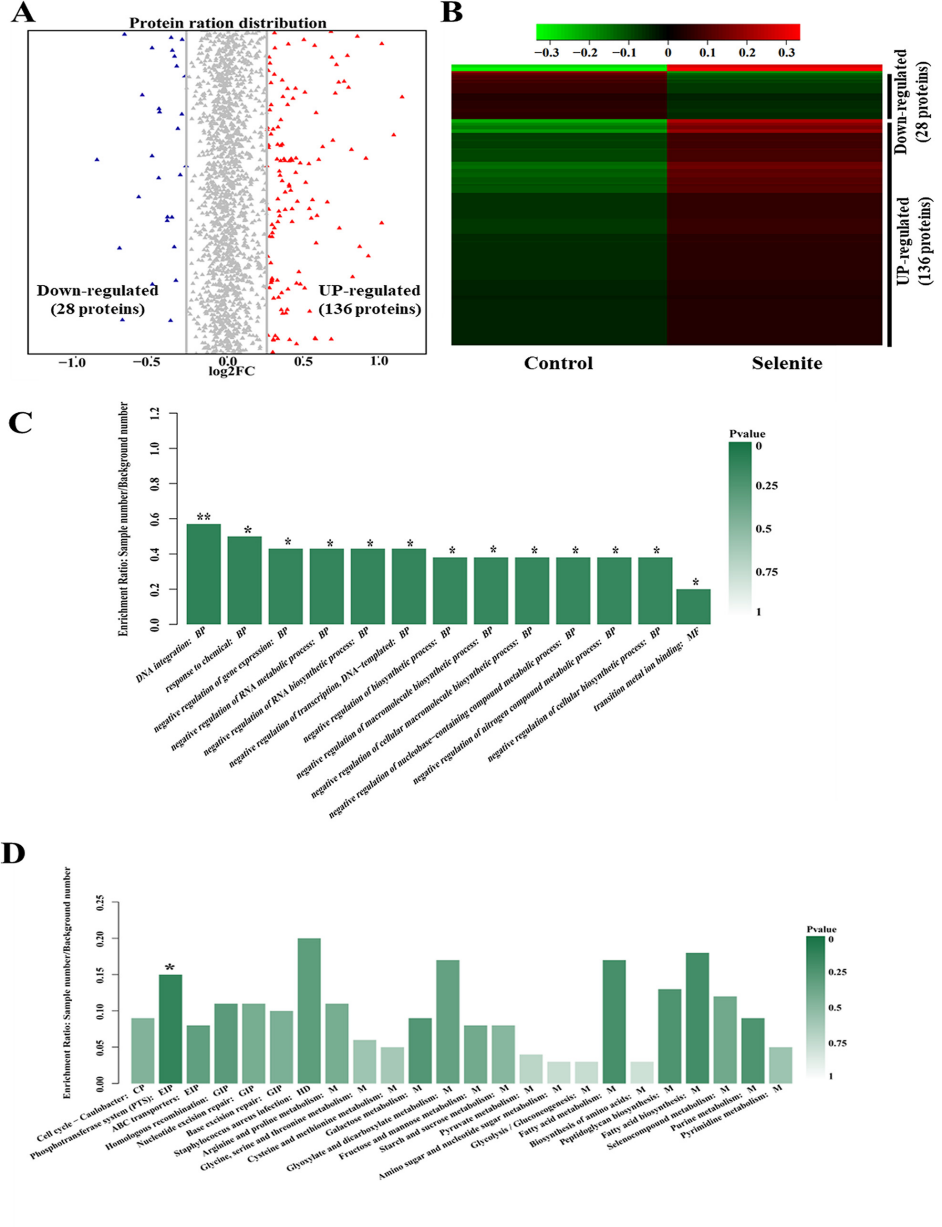
Identification of differentially expressed proteins in *L. casei* ATCC 393 exposed to sodium selenite. (A) Volcano plot showing up- and downregulated protein members. (B) Cluster heatmap of differentially expressed proteins. (C) GO functional classification of differentially expressed proteins. BP: biological process, CC: cell component, MF: molecular function. (D) KEGG pathway annotation of differentially expressed proteins. *N* = 3.

Subsequently, the differentially expressed proteins were identified by applying thresholds for fold change (>1.2 or <0.83) and *P* value (<0.05). A volcano plot and heatmap were utilized to showcase the potential biomarkers that may account for the disparities between the control and selenite-treated groups. As shown in Fig. 4A and B, a total of 164 (136 upregulated and 28 downregulated) differentially expressed proteins were screened. Differentially expressed proteins with identifying information are shown in Table 2. GO functional significance enrichment analysis was performed on the differential proteins, and it was found that most of the differential metabolites were enriched in the BP, among which DNA integration and response to chemical had the highest enrichment rates and significant differences (Fig. 4C). The KEGG pathways enrichment analysis of differentially expressed proteins resulted in the assignment of them to 24 KEGG pathways, of which the significantly different pathway was phosphotransferase system (Fig. 4D).

**TABLE 2:** List of differentially expressed proteins^*a*^

No.	Protein	FC	Accession (UniProt database)	Trend
1	Putative secreted protein	4.392	S6C890	Up
2	Cell surface protein	3.631	S6CK43	Up
3	Glycosyl hydrolase	2.707	S6CK93	Up
4	Sugar ABC transporter substrate binding component	2.432	S6CK72	Up
5	Putative cell wall-associated hydrolase	2.365	S6BP29	Up
6	Transporter protein	2.032	S6CJW2	Up
7	Putative multidrug ABC transporter ATP-binding and permease components	2.031	S6C4X3	Up
8	Putative phage tail protein	1.89	S6C9Q1	Up
9	Putative transporter protein	1.836	S6C030	Up
10	ABC transporter ATP-binding component	1.817	S6CK26	Up
11	Lipoprotein	1.778	S6BW99	Up
12	Glycine cleavage system protein	1.768	S6C9Y6	Up
13	Phage tail component	1.745	S6CIY0	Up
14	Putative transcriptional regulator	1.736	S6CJM7	Up
15	ABC transporter ATP-binding component	1.673	S6C2D2	Up
16	Phage NTP-binding protein	1.668	S6CJ31	Up
17	Putative Glutaredoxin	1.651	S6CJG6	Up
18	Putative cell surface protein	1.642	S6CIS3	Up
19	Tyrosine recombinase XerC	1.624	S6CA67	Up
20	Oxidoreductase	1.617	S6CJL4	Up
21	Phage integrase	1.612	S6BQG1	Up
22	Arsenical resistance operon transacting repressor	1.61	S6CK81	Up
23	Phage antirepressor protein	1.522	S6C9N4	Up
24	Hypothetical phage protein	1.51	S6C5P1	Up
25	Amino acid ABC transporter permease component	1.504	S6C0H7	Up
26	PTS system sucrose-specific IIAB components	1.504	S6C890	Up
27	Endopeptidase	0.829	S6CJ91	Down
28	Phosphoribosylaminoimidazole-succinocarboxamide synthase	0.807	S6C937	Down
29	Primosomal protein N	0.798	S6CAJ6	Down
30	DNA topoisomerase 1	0.794	S6CJF4	Down
31	Putative hydrolase	0.786	S6CJM2	Down
32	N5-carboxyaminoimidazole ribonucleotide mutase	0.785	S6C024	Down
33	Glycosyltransferase	0.778	S6CJR8	Down
34	Transcriptional regulator	0.777	S6C5H8	Down
35	33 kDa chaperonin	0.774	S6CK17	Down
36	Competence protein	0.772	S6BS42	Down
37	Pyruvate oxidase	0.761	S6C5I8	Down
38	Initiation-control protein YabA	0.732	S6CBJ7	Down
39	Transcriptional repressor NrdR	0.731	S6C908	Down
40	Transcriptional regulator	0.71	S6BVQ1	Down
41	UvrABC system protein A	0.709	S6C9S7	Down
42	Putative hydrolase	0.709	S6CJT3	Down
43	ATP-dependent Clp protease ATP-binding subunit ClpX	0.667	S6C7P1	Down
44	Formamidopyrimidine-DNA glycosylase	0.611	S6BZY4	Down
45	Putative transcriptional regulator	0.551	S6C9N1	Down
46	Esterase	0.424	S6C9N0	Down

a The differentially expressed proteins were identified by applying thresholds for fold change (>1.2 or < 0.83) and *P* value (<0.05). FC: fold change.

### Reduction mechanism of selenite to SeNPs by *L. casei* ATCC 393.

**FIG 5:**
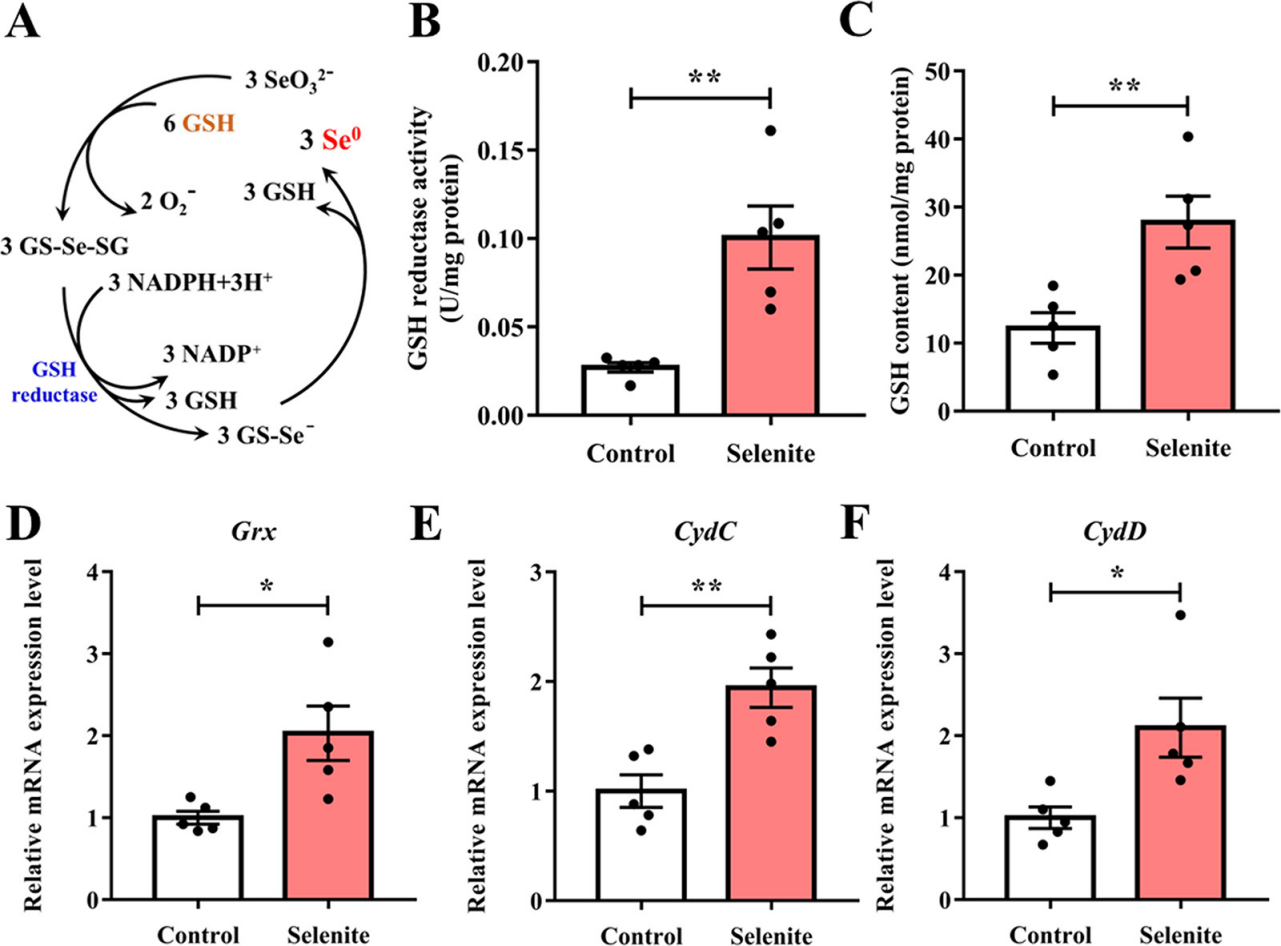
Reduction mechanism of selenite to SeNPs by *L. casei* ATCC 393. (A) Mechanism diagram of GSH-mediated Painter-type reaction. Selenite reacts with bacterial GSH to generate selenodiglutathione (GS-Se-SG), and GS-Se-SG is further reduced to selenopersulfide of glutathione (GS-Se-) by the catalysis of GSH reductase. Since GS-Se- is not stable, it will continue to hydrolyze to generate relatively stable Se^0^ and GSH. (B) GSH reductase activity in *L. casei* ATCC 393 exposed to sodium selenite. (C) GSH content in *L. casei* ATCC 393 exposed to sodium selenite. (D) The mRNA expression level of glutaredoxin (Grx) in *L. casei* ATCC 393 exposed to sodium selenite. (E) The mRNA expression level of *CydC* in *L. casei* ATCC 393 exposed to sodium selenite. (F) The mRNA expression level of *CydD* in *L. casei* ATCC 393 exposed to sodium selenite. Data are expressed as mean ± SEM. *N* = 5. *, *P < *0.05; **, *P < *0.01.

After careful review of the differentially expressed protein list, we observed a 1.651-fold increase in glutaredoxin (Grx) expression in bacteria after selenite treatment (Table 2). The Painter-type reaction in the cytoplasm is one of the mechanisms for the reduction of selenite to Se^0^ (Fig. 5A) The increased expression of Grx implies that *L. casei* ATCC 393 may also reduce selenite to elemental Se^0^ via the glutathione (GSH)-mediated Painter-type reaction pathway. Follow-up verification experiments also further confirmed the correctness of the conjecture. Our results showed that selenite treatment significantly increased *Grx* mRNA expression level (Fig. 5B), GSH content (Fig. 5C) and GSH reductase activity (Fig. 5D) in *L. casei* ATCC 393.

In addition, we found that GSH in the MRS broth medium was as high as 132 ± 21 μM, which provided conditions for selenite reduction. Moreover, an increase in the expression of proteins related to ATP-binding cassette (ABC) type transporter was also observed in the differentially expressed protein list (Table 2), including S6CK72 (2.432-fold), S6C4X3 (2.031-fold), S6CK26 (1.817-fold), S6C2D2 (1.673-fold) and S6C0H7 (1.504-fold). Furthermore, compared with the control group, selenite treatment significantly increased the *CydC* and *CydD* (putative cysteine and GSH importer) mRNA expression levels of *L. casei* ATCC 393 (Fig. 5E and F).

### Effect of GSH on the reduction of selenite by *L. casei* ATCC 393.

**FIG 6:**
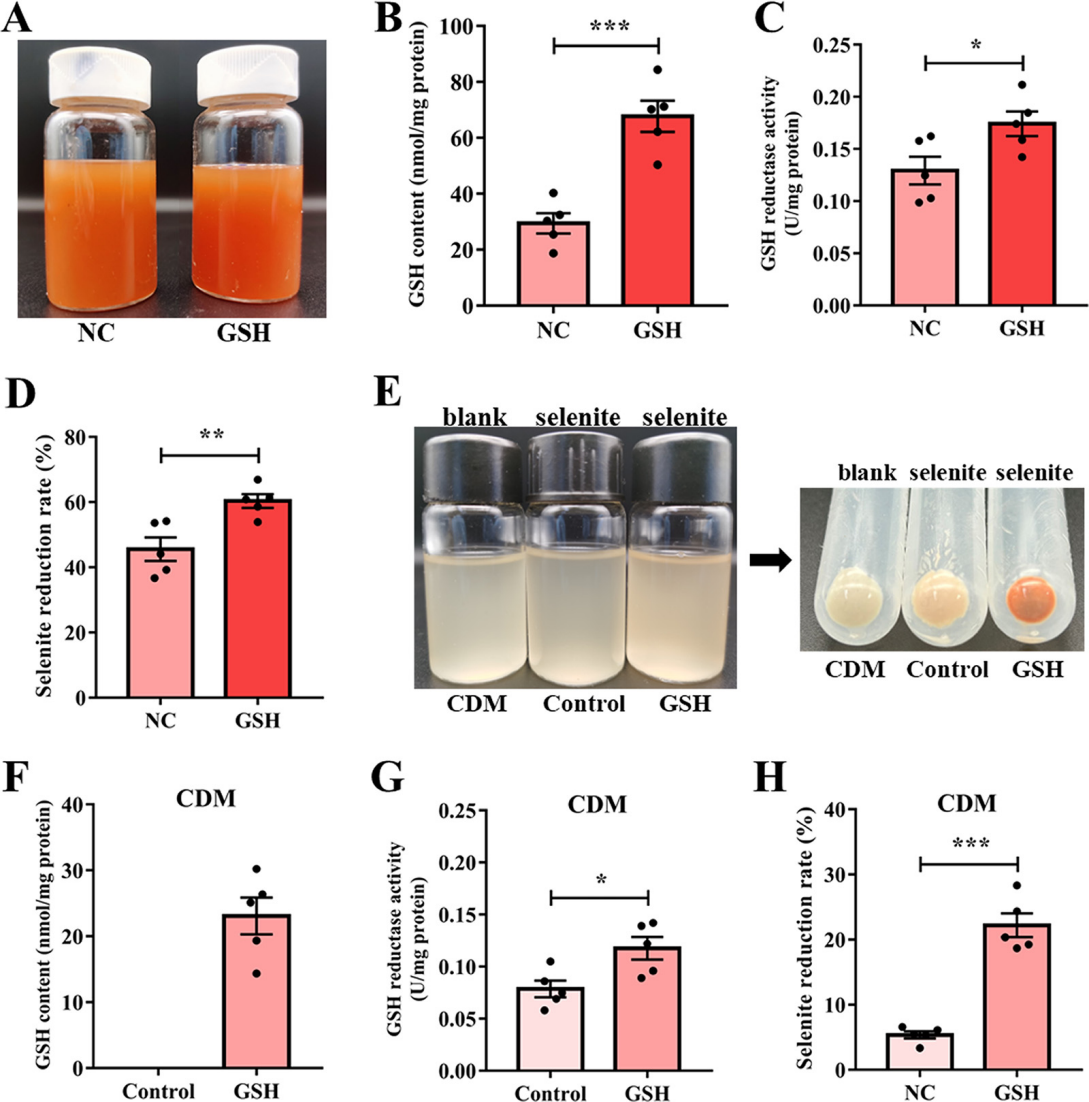
Effect of GSH on the reduction of selenite by *L. casei* ATCC 393. (A) Color change of *L. casei* ATCC 393 cultures (contains sodium selenite) in MRS medium supplemented with 2 mM GSH. (B) GSH content (MRS medium). (C) GSH reductase activity (MRS medium). (D) Reduction rate of selenite (MRS medium). (E) Color change of *L. casei* ATCC 393 cultures in CDM. In the exponential phase, 4 mM sodium selenite and/or 2 mM GSH were added to the CDM and the culture was continued at 37°C for 24 h. (F) GSH content (CDM). (G) GSH reductase activity (CDM). (H) Reduction rate of selenite (CDM). Data are expressed as mean ± SEM. *N* = 5. *, *P < *0.05; **, *P < *0.01; ***, *P < *0.001.

As shown in Fig. 6 A, compared with the negative control group (NC group, contains only background GSH), the addition of 2 mM GSH to the MRS medium resulted in a brighter red color, implying that more selenite was reduced and more SeNPs were synthesized. In addition, adding additional GSH to the culture medium significantly increased the GSH content (Fig. 6B) and GSH reductase activity (Fig. 6C) in *L. casei* ATCC 393. It is worth noting that the reduction rate of selenite increased by 17.25% (Fig. 6D). Furthermore, the role of GSH in the reduction of selenite by *L. casei* ATCC 393 was further explored using chemically defined medium (CDM) without GSH instead of MRS medium, and it was found that the cultures in the selenite group did not show a significant red color compared to the blank group and the rate of selenite reduction was only 5.36 ± 0.55%, whereas the addition of GSH to CDM resulted in the appearance of red colored organisms (Fig. 6E). Moreover, supplementation of GSH to CDM significantly increased the GSH content and GSH reductase activity, as well as the efficiency of selenite reduction in *L. casei* ATCC 393 compared to the control group in CDM (Fig. 6F–H). The above data suggest that the GSH-mediated Painter-type reaction may be the main pathway of selenite reduction in *L. casei* ATCC 393. Moreover, the presence of GSH in *L. casei* ATCC 393 can improve the reduction efficiency of selenite and the yield of SeNPs, which provides ideas for rapid bioremediation of Se contamination and efficient synthesis of SeNPs.

### Effect of oxidoreductase additives and inhibitors on the reduction of selenite.

**FIG 7:**
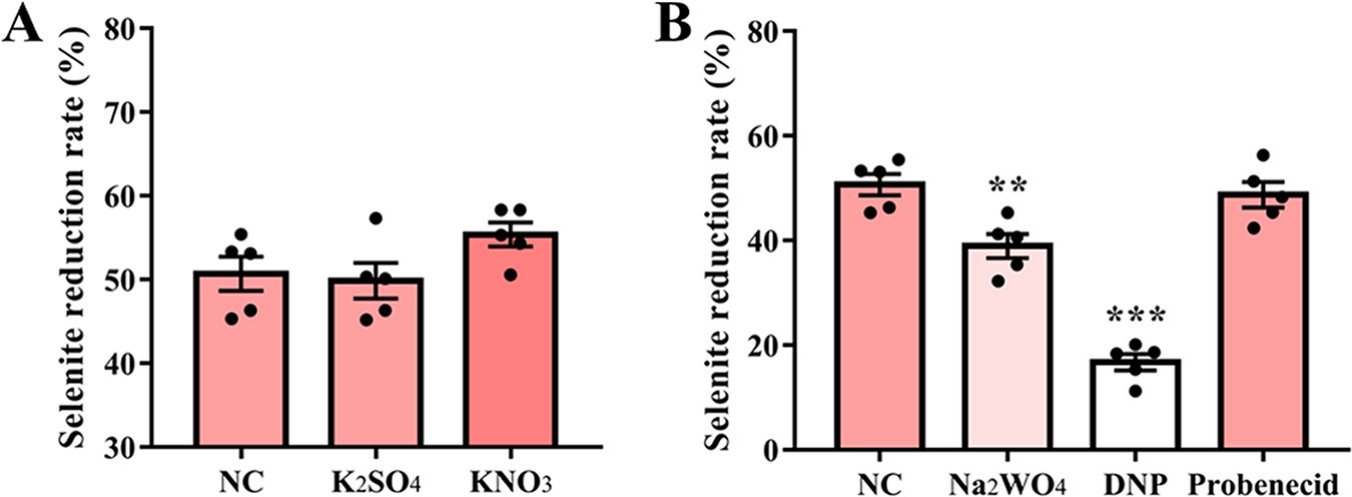
Effect of oxidoreductase additives and inhibitors on the reduction of selenite. (A) Effect of potassium sulfate and potassium nitrate on selenite reduction. (B) Effects of DNP and probenecid as transport inhibitors and sodium tungstate as a nitrate reductase inhibitor on selenite reduction. NC: no additives or inhibitors added. Data are expressed as mean ± SEM. *n* = 5. **, *P < *0.01; ***, *P < *0.001.

The addition of potassium nitrate (nitrate reductase activator) increased the reduction rate of sodium selenite in *L. casei* ATCC 393 from 50.68 ± 2.0% to 55.37 ± 1.44% compared with the NC group, while potassium sulfate (sulfate reductase activator) had almost no effect on the reduction rate (Fig. 7A). In addition, 2,4-dinitrophenol (DNP, nitrate transporter protein inhibitor) and sodium tungstate (nitrate reductase inhibitor) significantly inhibited the reduction of sodium selenite, while probenecid (sulfate transporter protein inhibitor) had no significant effect on the reduction rate (Fig. 7B).

## DISCUSSION

*L. casei* is one of the most widely studied and applied food-grade LABs that does not contain any toxic substances (29). In this study, we have discovered *L. casei* ATCC 393 possesses the capability to transform selenite to elemental Se^0^ (the colonies and cultures turned red when selenite was added) and form SeNPs with a particle size of 65 ± 22 nm. In addition, *L. casei* ATCC 393 can reduce 4 mM sodium selenite by 95% within 72 h. Moreover, we found that the reduction efficiency of *L. casei* ATCC 393 to selenite decreased with increasing selenite concentration, suggesting that selenite has a toxic effect on *L. casei* ATCC 393. The toxic effects of selenite on bacterial growth have been reported in many bacteria (32). The reduction of selenite to elemental Se^0^ may be the detoxification effect of the strain (33).

Based on LC-MS/MS analysis, we found that the purified SeNPs were covered with proteins with molecular weights of 11 to 17 kDa, which were 50S ribosomal proteins L7/L12. Microbially produced proteins, polysaccharides, or extracellular polymers interact with elemental Se^0^ and act as a corona for nanoparticles during the formation of SeNPs, which enhances their colloidal stability, influences their interactions with metal ions, and impacts their mobility (34–36). Biogenic SeNPs possess natural coating of biomolecules, which imparts stability and prevents aggregation over time (37). SeNPs with high stability can exhibit their inherent advantages in biomedical and industrial nanomaterials.

SeNPs were mainly produced intracellularly, which indicated that the selenite reduction process was mainly through the associated proteins and enzymes in the cytoplasm (37). Few studies have reported information on how selenite reaches the cytoplasm. Our proteomic analysis found a significant increase in the expression of proteins related to ABC transporters. ABC transporters, which represent one of the oldest and largest protein superfamilies found in all living organisms, facilitate transportation by utilizing ATP binding, hydrolysis, and substrate translocation across membranes. These transporters have a broad range of substrates (38). Which type of ABC transporter protein is involved in the selenite transport of *L. casei* ATCC 393 remains to be further studied. Studies have shown that the transport of selenite into reducing bacterial cells may be carried out through sulfate transporters or nitrate transporters, both of which belong to the ABC transporters (39, 40). In this study, by adding DNP (nitrate transporter protein inhibitor) and probenecid (sulfate transporter protein inhibitor) to the reduction system, it was found that DNP significantly inhibited selenite reduction, suggesting that in *L. casei* ATCC 393, nitrate transporter protein may be the protein mainly responsible for selenite transport. In addition, Pinel-Cabello et al. (14) identified key outer membrane transporters involved in selenite transport in strain Stenotrophomonas bentonitica BII-R7, such as RND lipoprotein NodT, TonB-dependent transporters, metal transporters or porins.

The reduction of Se by microorganisms mainly includes assimilation reduction and dissimilatory reduction. Bacteria with the potential ability to assimilate Se account for about 33% of the total bacteria, and can assimilation reduce Se to Se-containing amino acids, such as selenomethionine (Se-Met), selenocysteine (SeCys), and selenocystine (SeCys2); these amino acids are further involved in the synthesis of selenoproteins (41). The dissimilatory reduction of Se by microorganisms mainly refers to the reduction of selenate and selenite to Se^0^ (42). There are many hypotheses about the pathway of bacterial reduction of selenite. Song et al. (11) found that Enterobacter cloacae Z0206 was able to efficiently reduce selenite to Se^0^ using fumarate reductase and subsequently synthesize SeNPs. Nitrite reductase can be involved in the reduction of selenite in Thauera selenatis and *Rhizohiuna selenitireducens* B1, respectively (43, 44). Sulfite reductase is involved in selenite reduction in Providencia rettgeri HF16-A (16). In addition, succinate dehydrogenase (18), fumarate reductase (45), arsenate reductase (46), chromate/selenite reductase CsrF (47), hydrogenase I (48), and thioredoxin reductase (49) were also involved in selenite reduction in their respective strains. The most typical pathway for bacterial reduction of selenite is the Painter-type reaction (GSH reduction). Selenite undergoes a reaction with GSH resulting in the formation of GS-Se-SG. Subsequently, NADPH and GSH reductase further reduce GS-Se-SG to GS-Se-. As an unstable intermediate, GS-Se- undergoes hydrolysis reaction, leading to the formation of Se^0^ and reduced GSH (50). In this study, proteomic analysis found a 1.651-fold increase in Grx expression in bacteria after selenite treatment, which is dependent on reduced GSH as a cofactor for its activity (GSH–Grx-dependent reduction system). Grx is a widely distributed, heat-stable, small-sized oxidoreductase that is believed to be involved in various functions such as deoxyribonucleotide synthesis, repairing oxidatively damaged proteins, protein folding, and metabolism of sulfur (51). Studies have shown that GSH can promote the conversion of selenite to Se^0^ in microorganisms *via* the Painter-type reaction pathway (15). Significant increases in Grx mRNA expression level, GSH content, and GSH reductase activity in *L. casei* ATCC 393 also further confirmed that *L. casei* ATCC 393 may undergo selenite reduction via GSH-mediated Painter-type reaction pathway. In addition, the activation of the pentose phosphate pathway serves to generate more reducing power (specifically, NADPH) under selenite stress. This NADPH acts as an electron donor in the reduction of GS-Se-SG to GS-Se- by the GSH reductase and is essential for subsequent selenite reduction and SeNP formation reactions.

GSH is widely distributed in eukaryotes and Gram-negative prokaryotes, but most Gram-positive prokaryotes, including *L. casei* ATCC 393, do not have γ-glutamylcystiene synthetase (GshA) and glutathione synthetase (GshB) genes for the biosynthesis of GSH (52). However, Gram-positive bacteria can transport GSH from the medium via the ABC transporter (53). GSH transport in prokaryotes is known to be carried out by a heterodimeric ABC-type transporter CydDC (consisting of two subunits CydC and CydD, putative cysteine and glutathione importer) (54). In this study, we found that GSH in the MRS broth medium was as high as 132 ± 21 μM, and selenite treatment significantly increased the *CydC* and *CydD* mRNA expression level of *L. casei* ATCC 393. Furthermore, additional supplementation of GSH in the medium significantly increased the GSH content and GSH reductase activity, and the reduction rate of selenite increased by 17.25%. Furthermore, we used a CDM (without GSH) to further validate our hypothesis. The results showed that when sodium selenite was added to the CDM, we hardly observed any significant appearance of red bacterial cells, with a reduced rate of only 5.36%. However, after adding extra GSH, the bacterial cells turned noticeably red, and the content of GSH and the activity of GSH reductase inside the bacterial cells were significantly increased, leading to an increased reduction rate of 22.19%. These results further suggest that the GSH-mediated Painter-type reaction is the main pathway for selenite reduction in *L. casei* ATCC 393, but there are other reduction pathways that may also be involved in selenite reduction.

In addition to the GSH system, sulfate reductase and nitrate reductase may also catalyze the formation of Se^0^ from selenite (55). Indeed, proteomic data revealed a 1.617-fold increase in the expression of oxidoreductases in *L. casei* ATCC 393 after the addition of sodium selenite. Sulfate and nitrate can promote the reducing activity of sulfate reductase and nitrate reductase, respectively (17). Therefore, adding these substances during the reduction of sodium selenite by *L. casei* ATCC 393 and observing their impact on the reduction efficiency can help infer the types of enzymes involved in the reduction of sodium selenite by *L. casei* ATCC 393. Here, when potassium sulfate was added to the culture medium, the reduction rate was 50.68%, which showed little difference compared to the blank control (49.84%). However, when potassium nitrate was added, the reduction efficiency increased to 55.37%. Moreover, the addition of nitrate reductase inhibitor sodium tungstate to the reduction system can significantly inhibit the reduction of selenite. This result suggests that in the *L. casei* ATCC 393, the enzyme responsible for reducing sodium selenite to Se^0^ includes not only GSH reductase but also nitrate reductase.

Improving cellular tolerance by excreting harmful substances is a common strategy for bacteria to cope with environmental toxins such as heavy metals and antibiotics (56). It was found by TEM that SeNPs can exist on the cell surface, cytoplasm, and culture medium. The process of reducing selenite to Se^0^ is generally completed in the cytoplasm, so to avoid the damage of the formed SeNPs to the cells, Se-reducing microorganisms must have a secretion mechanism to transfer them outside the cell. The reduction of selenite by Thauera selenatis is carried out by selenate reductase (Ser), in which SefA has the function of SeNPs aggregation and plays an important role in the secretion of SeNPs (57, 58). From the proteomics data of this study, the expression of cell wall-associated hydrolase in *L. casei* ATCC 393 was upregulated 2.365-fold, which may be a self-rescue measure for bacteria in the face of selenite pressure. Cell wall-associated hydrolases are a class of enzymes that are involved in the construction, remodeling, and degradation of peptidoglycan within the bacterial cell wall. They serve critical functions in cell wall metabolism, bacteriolysis, expansion of the environmental niche, and defense against environmental toxins and stress (59). *L. casei* ATCC 393 may help SeNPs to be released extracellularly by upregulating the expression of cell wall-associated hydrolase. In addition, the release of intracellular nutrients may also provide energy and nutrients for other normal bacteria. However, the current research on the exudation mechanism of SeNPs is limited and our understanding is also very limited. More research is needed to further explore this topic.

When bacteria are exposed to environmental toxicants, the presence of toxicants may affect the metabolic pathways of bacteria, resulting in a decrease in the rate of energy metabolism (60). To compensate for the decreased rate of energy metabolism, bacteria increase the expression of energy metabolism-related proteins that may be involved in metabolic pathways, including sugar catabolism, oxidative phosphorylation, and amino acid metabolism to increase the rate of metabolic pathways (61, 62). Here, we observed an increase in the expression of glycosyl hydrolases, glycine cleavage system proteins and phosphotransferase system (PTS) system sucrose-specific IIAB components from proteomics, and the functions of these proteins are involved in the energy metabolism of bacteria. The PTS system is a major carbohydrate active transport system, and the PTS system sucrose-specific IIAB components may be specific for the transport of glucose and sucrose (63). Glycosyl hydrolases are a group of enzymes that can break down the glycosidic bond between sugar molecules, including glucose, to release simple sugars, providing nutrients and energy for bacteria (64). The glycine cleavage system is a metabolic pathway widely present in bacteria, which releases energy by converting glycine into pyruvate and formate, providing energy and substrate metabolites for bacteria. In addition, in some special environments, the glycine cleavage system may also be involved in the adaptation of bacteria to environmental stress (65). The upregulation of protein expression mentioned above may be a self-protective measure of *L. casei* ATCC 393 in response to selenite stress.

### Conclusions.

**FIG 8:**
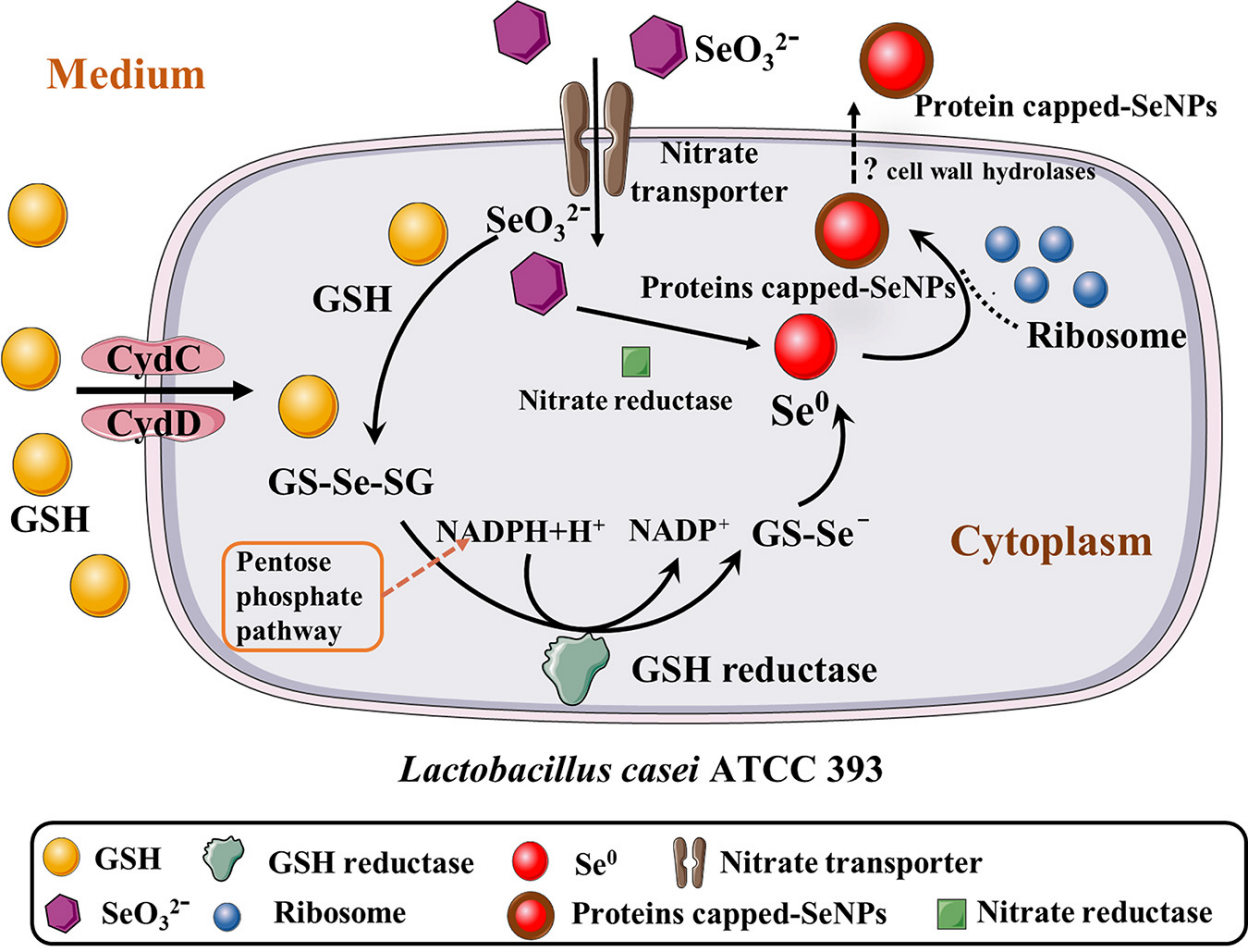
Mechanism and schematic model of selenite reduction by food-grade LAB *L. casei* ATCC 393. Dotted lines show yet unidentified processes.

Food-grade probiotic *L. casei* ATCC 393 can reduce toxic selenite and form 50S ribosomal protein L7/L12 capped-SeNPs with a particle size of 65 ± 22 nm. Furthermore, the GSH-mediated Painter-type reaction may be the main pathway of selenite reduction by *L. casei* ATCC 393. GSH can enter the cytoplasm through CydC and CydD and react with selenite to generate SeNPs under the action of GSH reductase. In addition, nitrate reductase also participates in the reduction process of selenite, but it is not the primary factor (Fig. 8). Overall, this study provides an efficient and environmentally friendly biocatalyst for the bioremediation of Se contamination and the synthesized protein capped-SeNPs can also be economically applied to biomedicine, agriculture, biosensor.

## MATERIALS AND METHODS

### Strain and reagents.

*L. casei* ATCC 393 was purchased from the American Type Culture Collection. The *L. casei* ATCC 393 was activated and cultivated in MRS broth medium. Sodium selenite (cat. no. S5261) was obtained from Sigma-Aldrich (Saint Louis, MO, USA). Glutathione (GSH, cat. no. G8180) and GSH reductase activity detection kit (cat. no. BC1160) were purchased from Beijing Solarbio (Beijing, China). Total GSH assay kit (cat. no. S0052) was obtained from Beyotime (Shanghai, China). RNAex pro reagent (cat. no. AG21102) was obtained from Accurate Biology (Hunan, China). ABScript III RT Master Mix (cat. no. RK20429) and qPCR Mix (cat. no. RK21203) were obtained from ABclonal (Wuhan, China).

### Bacterial culture and selenite reduction identification.

*L. casei* ATCC 393 was cultured in MRS broth medium at 37°C without shaking and incubated for 12 h. Next, 100 μL of 10^−6^ dilution of the culture solution was inoculated onto MRS plates with or without 1 mM sodium selenite and then cultured at 37°C for 72 h. Single red colonies (indicating selenite reduction and Se^0^ formation) were recultured in MRS medium to obtain sodium selenite-tolerant strain for subsequent experiments. To further verify the effect of *L. casei* ATCC 393 on reducing selenite, control treatments without sodium selenite and/or bacteria, and supernatant of *L. casei* ATCC 393 with sodium selenite were also established.

### Bacterial growth under selenite stress.

To determine the effect of selenite on the growth of *L. casei* ATCC 393, overnight-grown bacterial culture was adjusted to an optical density at 600 nm (OD_600_) of 0.6 and inoculated (1% inoculum size) to the MRS broth medium. Then, different concentrations (0 mM, 0.5 mM, 1 mM, 2 mM, 4 mM, 8 mM, and 16 mM) of sodium selenite were added at different growth stages (lag phase: 3 h, exponential phase: 12 h, stationary phase: 27 h) of the bacteria, respectively. During the 72 h of cocultivation, the bacterial culture was collected at regular intervals. After the total bacterial protein was extracted, the concentration of bacterial protein was detected to characterize the number of bacterial cells.

### Selenite biotransformation assays.

To determine the reduction efficiency of *L. casei* ATCC 393 to selenite. During the 72 h of cocultivation, the bacterial culture supernatant was collected at regular intervals, and the Se content in the medium was detected by inductively coupled plasma-mass spectrometry (ICP-MS) (1). The Se biotransformation ratio by the biomass was calculated by determining the difference in Se concentration before and after culture.

### Localization and characterization of SeNPs.

Bacteria grown overnight were adjusted to OD_600_ = 0.6 and inoculated into MRS broth medium at 1% inoculum. After 12 h (exponential phase), 4 mM sodium selenite was added to the medium and the culture was continued at 37°C for 72 h. The location of SeNPs within the *L. casei* ATCC 393 was determined using SEM-EDS and TEM-EDX analysis. In addition, the valence state of Se in SeNPs was analyzed by X-ray photoelectron spectroscopy (XPS). Surface proteins of SeNPs were identified by LC-MS/MS, and colloidal stability of SeNPs by was evaluated by detecting the zeta potential.

**(i) SEM-EDS analysis.** The 1 mL of bacterial culture was centrifuged at 8,000 rpm for 5 min to collect bacteria. The bacteria were then resuspended in prechilled fixation solution (4% glutaraldehyde in 0.01 M PBS, pH 7.4) and fixed overnight at 4°C. After fixation, bacteria were rinsed twice with PBS for 10 min each time. Dehydration was subsequently performed using ethanol gradients of different concentrations (30%, 50%, 70%, 90%, and 100%) for 10 min each. Finally, isoamyl acetate was used to displace the ethanol. After drying and sputter-coating, morphology was observed by SEM (VEGA 3 SBH, Czech). Elemental composition maps of selected areas were analyzed with the EDS (Oxford X-act, U.K.) system.

**(ii) TEM-EDX analysis.** After processing the fixed samples using standard procedures, such as staining, dehydration, embedding, and slicing into ultrathin sections, the ultrastructure of SeNPs was observed using TEM (JEM-F200, Japan), and the elemental composition of SeNPs was analyzed using EDX (JED-2300T, Japan).

**(iii) XPS analysis.** The produced SeNPs were purified according to our previously established method (27). After the SeNPs were freeze-dried, the valence state of Se was analyzed by XPS (Axis Ultra DLD, Japan).

**(iv) Identification of surface proteins of SeNPs by LC-MS/MS.** Isolated biogenic SeNPs were analyzed by SDS-PAGE. Then the main protein band was collected by the Ultimate 3000 system (Thermo Fisher Scientific, USA) coupled with a Q Exactive Hybrid Quadrupole-Orbitrap mass spectrometer (Thermo Fisher Scientific, USA) to analyze the protein type on the surface of biogenic SeNPs. The raw mass spectrometry (MS) files were analyzed and searched against a target protein database that was based on the species of the samples, using MaxQuant version 1.6.2.10. The reference species was Lactobacillus casei. Corresponding details were shown in the supplement material.

**(v) Zeta potential.** After resuspending SeNPs in 0.01 M PBS (pH 7.4). Surface charge of the nanoparticles were measured using a Malvern Zetasizer Nano-ZS instrument.

### iTRAQ quantitative proteomics.

To identify the probable mechanism involved in the reduction of selenite by *L. casei* ATCC 393, the proteomic differences between *L. casei* ATCC 393 treated with or without sodium selenite were investigated using iTRAQ coupled with LC-MS/MS. Briefly, *L. casei* ATCC 393 cultures untreated and treated with 4 mM sodium selenite for 12 h were harvested by centrifugation at 5,000 rpm for 5 min at 4°C. After washing twice with 10 mM Tris-HCl (pH 7.5), the whole-cell protein was separated from bacterial precipitation using a total bacterial protein extraction assay kit with protease inhibitor. Protein samples were subjected to reductive alkylation after reaching the standard by SDS-PAGE analysis and bicinchoninic acid (BCA) protein concentration assay. An equal amount of protein was then digested with Trypsin and the peptides were labeled with iTRAQ reagent. After mixing the labeled peptides in equal amounts, the mixed peptides were preseparated by a C18 solid-phase column, and finally detected by LC-MS/MS. Corresponding details were shown in supplement material.

### mRNA expression analysis of the key proteins involved in the synthesis of SeNPs.

Total RNA was extracted from bacteria using the RNAex pro reagent. Next, 1 μg mRNA of satisfactory quality was selected for reverse transcription to obtain cDNA, which was subsequently subjected to qPCR. The oligonucleotide primers for target genes and a housekeeping gene (16S rRNA) are listed in Table S1. The relative mRNA abundance of the selected genes was calculated using the 2^−ΔΔCt^ method.

### Determination of GSH concentrations.

The GSH content in MRS medium and bacteria were determined using the total GSH assay kit (Beyotime, cat. no. S0052) according to the manufacturer’s instructions.

### GSH reductase activity assay.

The GSH reductase activity in bacteria was determined using GSH reductase activity detection kit (Solarbio, cat. no. BC1160) according to the manufacturer’s instructions.

### Effect of GSH on the reduction of selenite by *L. casei* ATCC 393.

Bacteria grown overnight were adjusted to OD_600_ = 0.6 and inoculated into MRS broth medium at 1% inoculum. After 12 h, 4 mM sodium selenite and 2 mM GSH were added to the medium and the culture was continued at 37°C for 24 h. Media without GSH served as negative control (NC). In addition, after subculturing in MRS broth, *L. casei* ATCC 393 were inoculated at 1% in CDM (without GSH) as described by a previous study with slight modifications (66), to further explore the role of GSH in selenite reduction. In the exponential phase, 4 mM sodium selenite and/or 2 mM GSH were added to the CDM, and the culture was continued at 37°C for 24 h. After the above treatments, the GSH content, GSH reductase activity, and selenite reduction rate in bacteria were detected.

### Effect of oxidoreductase additives and inhibitors on the reduction of selenite.

Additives (potassium sulfate and potassium nitrate, 2 mM) were added to the MRS medium with 4 mM sodium selenite in the exponential phase, and the culture was continued at 37°C for 24 h. Media without additives served as NC. Similarly, DNP (1 mM) and probenecid (2 mM) acted as transport inhibitors to explore the transport of selenite in *L. casei* ATCC 393. Sodium tungstate (30 mM) was used as a nitrate reductase inhibitor to study the selenite reduction pathway of *L. casei* ATCC 393. After the above treatments, the selenite reduction rate in bacteria was detected.

### Statistical analysis.

The data are presented as the mean ± SEM. Statistical analysis was carried out utilizing the GraphPad Prism 7.0 software. The significance of differences between two groups was determined using Student's *t* test. One-way analysis of variance (ANOVA) followed by a least significant difference (LSD) multiple-comparison test was utilized to establish statistical significance for multiple comparisons. *P* < 0.05 was considered statistically significant.

### Data availability.

The proteome data can be found at the Mendeley Data (https://data.mendeley.com/datasets/swbfnyd2kn).
