# Supplementary material for: Selenite Bioremediation by Food-Grade Probiotic Lactobacillus casei ATCC 393: Insights from Proteomics Analysis
Source: Microbiol Spectr. 2023 May 23;11(3):e00659-23. doi: 10.1128/spectrum.00659-23 (PMC10269480; doi:10.1128/spectrum.00659-23)
Supplement: Supplemental file 1 — Supplemental material. Download spectrum.00659-23-s0001.pdf, PDF file, 0.3 MB [file spectrum.00659-23-s0001.pdf]

Supplementary information

Selenite bioremediation by food-grade probiotic *Lactobacillus casei* ATCC 393: insights from proteomics analysis

Lei Qiao, Xina Dou, Xiaofan Song, Jiajing Chang, Xiaonan Zeng,

Lixu Zhu, Chunlan Xu*

School of Life Sciences, Northwestern Polytechnical University, Xi'an, Shaanxi,

710072, China

---

* Corresponding author: Chunlan Xu, Professor, School of Life Sciences, Northwestern Polytechnical University, 127 Youyixi Road Xi'an, Shaanxi, 710072, China. E-mail: clxu@nwpu.edu.cn Telephone: +86-29-88460543, Fax: +86-29-88460332

Supplementary Methods

1. Identification of surface proteins of SeNPs by liquid chromatography–tandem mass spectrometric (LC-MS/MS)

Isolated biogenic SeNPs was analyzed by SDS-PAGE. Then the main protein band was collected. Ultimate 3000 system (ThermoFisher Scientific, USA) coupled to a Q Exactive™ Hybrid Quadrupole-Orbitrap™ Mass Spectrometer (ThermoFisher Scientific, USA) was used to analyze the protein type on the surface of biogenic SeNPs. Chromatographic separations were performed on a reversedphase ReproSil-Pur C18-AQ resin (3 μm, 120 Å, Dr. Maisch GmbH, Germany). The mobile phase consisted of: (A) 0.1% formic acid in water and (B) 0.1% formic acid in acetonitrile. A linear binary mobile phase solvent gradient was used as follows: from 6% to 9% B for 8 min, from 9% to 14% B for 16 min, from 14% to 30% B for 36 min, from 30% to 40% B for 15 min and from 40% to 95% B for 3 min, eluting with 95% B for 7 min. The flow rate and injection volume were 600 nL/min and 5 μL, respectively. The mass spectrometer (MS) was operated using electrospray ionization (ESI) in positive- and negativeion modes. The raw MS files were analyzed and searched against target protein database based on the species of the samples using MaxQuant (1.6.2.10). The reference species is *Lactobacillus casei*. The parameters were set as follows: the protein modifications were carbamidomethylation (C) (fixed), oxidation (M) (variable); the enzyme specificity was set to trypsin; the maximum missed cleavages were set to 2; the precursor ion mass tolerance was set to 10 ppm, and MS/MS tolerance was 20 ppm. Only high confident identified peptides were chosen for following protein identification.

2. iTRAQ quantitative proteomics

2.1. Total protein extraction

L. casei ATCC 393 cultures untreated and treated with 4 mM sodium selenite for 12h were harvested by centrifugation at 5,000 rpm for 5 min at 4 °C. After washing twice with 10 mM Tris-HCl (pH 7.5), the whole-cell protein was separated from bacterial precipitation using a total bacterial protein extraction assay kit (Sangon Biotech, China) with protease inhibitor. The concentration of protein supernatant was determined by Bicinchoninic acid (BCA) method by BCA Protein Assay Kit (Pierce,Thermo, USA). Protein quantification was performed according to the kit protocol.

2.2. Protein Digestion and iTRAQ Labeling

Protein digestion was performed according to the standard procedure and the resulting peptide mixture was labeled using the 8-plex iTRAQ reagent (Applied Biosystems, 4390812) according to the manufacturer's instructions. Briefly, total protein (100 μg) taken from each sample was mixed with 100 μL of the lysate. TCEP (10 mM) was added and then it was stored at 37 °C. 60 min later, iodoacetamide (40 mM) was added and stored in dark at room temperature for 40 mins.

Six-fold volumes of cold acetone were added to precipitate protein at -20 °C for 4h. After centrifugation at 10000g at 4 °C for 20min, the pellet was re-suspended with 100μl 50mM riethylammonium bicarbonate (TEAB) buffer. Trypsin was added at 1:50 trypsin-to-protein mass ratio and incubated at 37 °C overnight. Then, one unit of iTRAQ reagent were thawed and reconstituted in 50 μL acetonitrile. After tagging for 2h at room temperature, hydroxylamine was added to react for 15min at room

temperature. Finally, all samples were pooled, desalted and vacuum-dried.

2.3. High pH RPLC Separation

The pooled samples were fractionated into fractions by ACQUITY Ultra Performance liquid chromatography (Waters, USA) with ACQUITY UPLC BEH C18 Column (1.7 µm, 2.1mm × 150 mm, Waters, USA) to increase proteomic depth. Briefly, peptides were first separated with a gradient of elution (Phase B: 5mM Ammonium hydroxide solution containing 80% acetonitrile, pH 10) over 48 min at a flowrate of 200µl/min. Twenty fractions were collected from each sample, which was subsequently pooled, resulting in ten total fractions per sample.

2.4. LC-MS/MS analysis

Labeled peptides were were analyzed by online nano flow liquid chromatography tandem mass spectrometry performed on an 9RKFSG2_NCS-3500R system (Thermo, USA) connected to a Q Exactive Plus quadrupole orbitrap mass spectrometer (Thermo, USA) through a nanoelectrospray ion source. Briefly, the C18-reversed phase column (75 µm x 25 cm , Thermo ,USA) as equilibrated with solvent A (A:2% formic acid with 0.1% formic acidr) and solvent B (B: 80% ACN with 0.1% formic acid).The peptides were eluted using the following gradient: 0-4 min, 0%-5% B; 4-66 min, 5%−23%B; 66-80 min, 23%−29% B; 80−89 min,29%−38% B; 89-91 min, 38-48% B; 91-92 min, 48-100% B; 92-105min, 100% B; 105-106min, 100-0% B) at a flow rate of 300nL/min . The Q Exactive Plus was operated in the data-dependent acquisition mode (DDA) to automatically switch between full scan MS and MS/MS acquisition. The survey of full scan MS spectra (m/z 350-1300) was acquired in the Orbitrap with 70000 resolution.

The automatic gain control (AGC) target at 3e6 and the maximum fill time was 20 ms. Then the top 20 most intense precursor ions were selected into collision cell for fragmentation by higher-energy collision dissociation (HCD). The MS/MS resolution was set at 35000 (at m/z 100), the automatic gain control (AGC) target at 1e5, the maximum fill time at 50 ms, and dynamic exclusion was 18 seconds.

2.5. Protein identification

The RAW data files were analyzed using ProteomeDiscoverer (Thermo Scientific, Version 2.1). Protein Database: uniprot-*Lactobacillus casei* subsp. casei ATCC 393 [219334] AND proteomeup000015560-2697s-20161205. The MS/MS search criteria were as follows: Mass tolerance of 10 ppm for MS and 0.05 Da for MS/MS Tolorance, trypsin as the enzyme with 2 missed cleavage allowed, carbamido methylation of cysteine and the iTRAQ of N-terminus and lysine side chains of peptides as fixed modification, and methionine oxidation as dynamic modifications, respectively. False discovery rate (FDR) of peptide identification was set as FDR ≤ 0.01. A minimum of one unique peptide identification was used to support protein identification.

2.6. Statistical analyses

Annotation of all identified proteins was performed using GO (http://www.blast2go.com/b2ghome; http://geneontology.org/) and KEGG pathway (http://www.genome.jp/kegg/). differentially expressed proteins were further used to for GO and KEGG enrichment analysis. Protein-protein interaction analysis was performed using the String v10.5.

Supplementary Figure

Fig. S1.

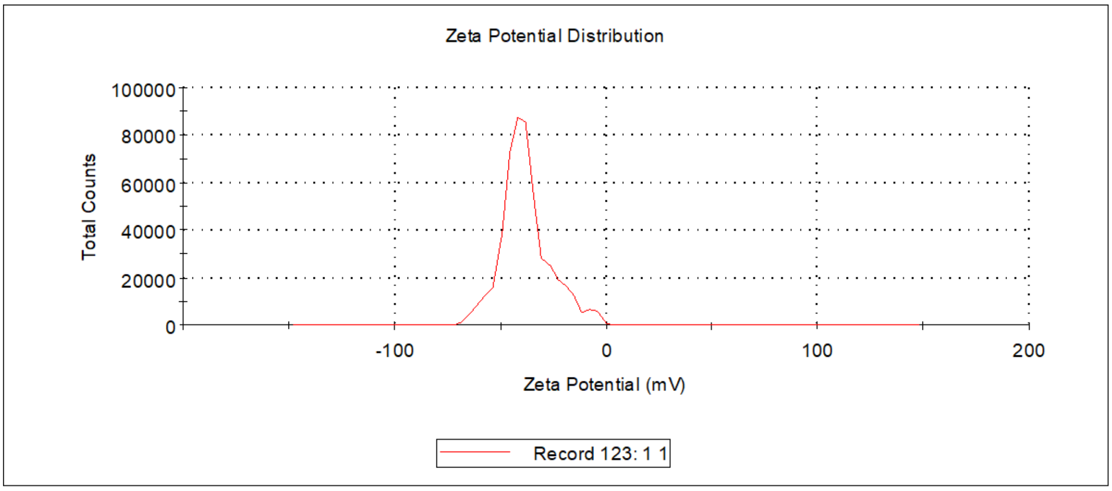

Fig. S1. Zeta potential distribution of SeNPs in 0.1 M PBS (pH 7.4).

Fig. S2.

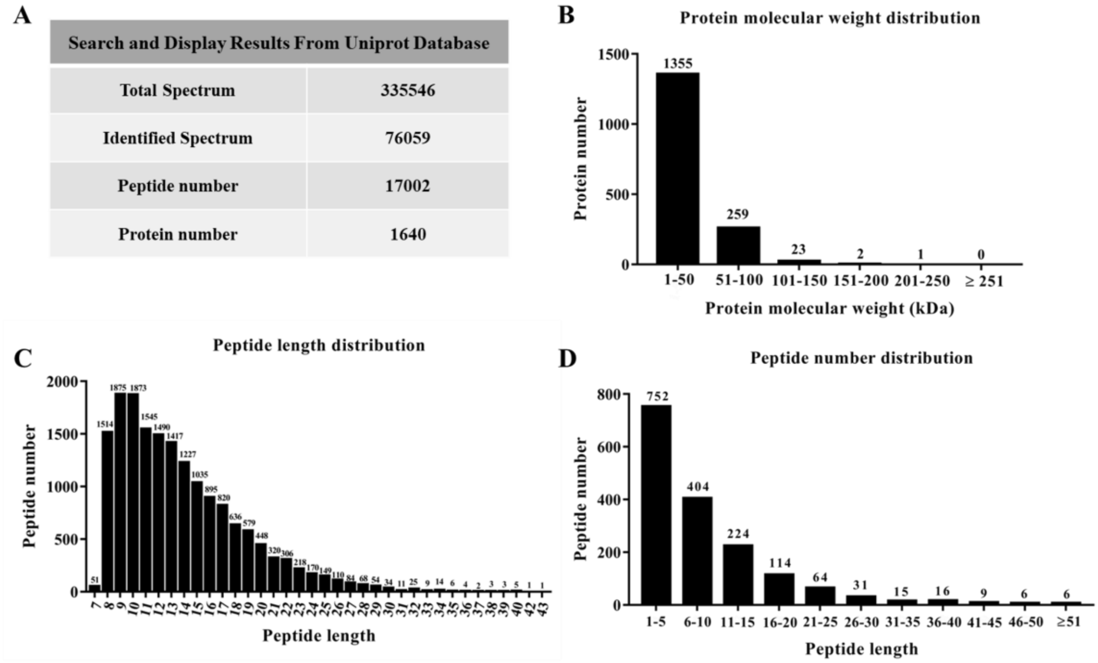

Fig. S2. iTRAQ quantitative proteomic identification information. **(A)** Search and display results from Uniprot database. **(B)** Protein molecular weight distribution. **(C)** Peptide length distributions. **(D)** Quantitative distribution of identified peptides.

Supplementary Table

Table S1 The primers and sequences of qPCR

Genes	Sequences (5'-3')
Grx	F: TTTCGGCAGGATGGTTATG<br>R: GCAAGTGCTTCGGGATGA
CydC	F: GCAACAAGGTCGTTTTCCCC<br>R: GCGGATGATTTTTCCGACCG
CydD	F: CCGTGCCGAAATCACGAATC<br>R: CGGTTGTCGCGGTTTTTCTT
16S rRNA	F: GCATTAAGCATTCCGCCTGG<br>R: TCATAAGGGTTGCGCTCGTT
